# Prevalence of fibromyalgia in asthma and its impact on asthma control: a case-control study

**DOI:** 10.1080/07853890.2025.2566392

**Published:** 2025-09-29

**Authors:** Suat Konuk, Alp Ozel, Emine Ozsari, Askin Nasircilar

**Affiliations:** a Department of Chest Diseases, Bolu Abant Izzet Baysal University, Bolu, Turkey; b Department of Physiotherapy and Rehabilitation, Bolu Abant Izzet Baysal University, Bolu, Turkey; c Department of Physical Medicine and Rehabilitation, Private Clinic, Bursa, Turkey

**Keywords:** Comorbidity, chronic pain, quality of life, self-assessment, central sensitization

## Abstract

**Background:**

Asthma and fibromyalgia are chronic conditions that significantly impair quality of life and share common inflammatory and neurophysiological mechanisms.

**Objective:**

This study aimed to investigate the prevalence of fibromyalgia in asthma patients and examine its impact on asthma control.

**Methods:**

In this case-control study, 120 patients diagnosed with asthma and 120 age- and sex-matched healthy controls were enrolled. Fibromyalgia was diagnosed based on the 2016 revised ACR criteria using the Widespread Pain Index (WPI) and Symptom Severity Scale (SSS). Asthma control was assessed using the Asthma Control Test (ACT). The relationship between fibromyalgia and asthma control was analyzed using independent t-tests, chi-square tests, logistic regression, and ROC analysis.

**Results:**

The prevalence of fibromyalgia in the asthma group was 16.7% compared to 3.3% in controls (*p* < 0.001). Asthma patients with fibromyalgia had significantly lower ACT scores than those without (18.2 ± 2.9 vs. 20.1 ± 2.4; *p* = 0.004). The proportion of patients with uncontrolled asthma was higher among those with fibromyalgia (60% vs. 35%). Logistic regression showed that fibromyalgia was associated with increased odds of uncontrolled asthma (OR = 2.16), although this was not statistically significant (*p* = 0.103). ACT scores showed no significant correlation with WPI or SSS. ROC analysis revealed that the ACT score had no discriminatory power in identifying fibromyalgia (AUC = 0.50).

**Conclusion:**

Fibromyalgia is a common comorbidity in asthma patients and may adversely affect perceived asthma control. However, the ACT score alone may not be a reliable indicator of fibromyalgia presence, suggesting the need for multidimensional assessment tools in routine asthma care.

## Introduction

Asthma is a chronic respiratory disease characterized by airway inflammation, bronchial hyperresponsiveness, and variable airflow limitation [1]. Fibromyalgia syndrome (FMS), on the other hand, is a chronic soft tissue disorder presenting with widespread musculoskeletal pain, fatigue, sleep disturbances, and cognitive dysfunction [2]. Both conditions significantly reduce quality of life, increase psychological burden, and are associated with increased healthcare utilization [3,4].

Recent studies have revealed a notable pathophysiological overlap between asthma and fibromyalgia. It has been suggested that neurogenic inflammation, central sensitization, and chronic pain syndromes may also occur in individuals with asthma [5,6]. Fibromyalgia is characterized by heightened sensitivity to pain in the central nervous system and may influence the pathogenesis of asthma through mechanisms involving proinflammatory cytokines (e.g. IL-6, TNF-α), neuropeptides (e.g. substance P), and hypothalamic-pituitary-adrenal (HPA) axis dysfunction [7,8]. Additionally, the high prevalence of psychological comorbidities such as anxiety and depression in both disorders is particularly noteworthy [9,10]. From a theoretical perspective, the intersection between asthma and fibromyalgia can be situated within the frameworks of central sensitization and neurogenic inflammation. These mechanisms mediated by cytokines, neuropeptides, and HPA axis dysregulation have been implicated in both conditions and offer a conceptual model for understanding the overlapping symptomatology and comorbid burden observed in affected individuals [7,11,12].

Clinical observations indicate that some patients with asthma exhibit fibromyalgia-like symptoms such as widespread pain, sleep disturbances, and fatigue [11,13]. However, the exact prevalence of fibromyalgia in asthma patients, its impact on asthma control, and whether this relationship is influenced by demographic factors such as age or sex remain unclear. Existing studies in the literature often have small sample sizes and rarely apply the 2016 American College of Rheumatology (ACR) criteria for diagnosing fibromyalgia [4]. Moreover, few studies have objectively examined the impact of fibromyalgia on asthma control using validated tools such as the Asthma Control Test (ACT).

The ACT is a commonly used self-reported instrument designed to assess asthma control, primarily focusing on respiratory symptoms. ecent studies indicate that coexisting conditions, especially chronic pain syndromes, depression, and anxiety, can have a negative impact on ACT scores. This implies that the ACT may reflect not only airway inflammation but also the individual’s overall health status and psychosocial burden. Therefore, understanding how comorbid conditions with systemic manifestations, such as fibromyalgia, influence asthma control as assessed by ACT is a subject worthy of further investigation.

Accordingly, this study aims to determine the prevalence of fibromyalgia in asthma patients diagnosed according to the 2016 ACR criteria, to evaluate the impact of fibromyalgia on asthma control as measured by ACT scores, and to investigate the role of demographic variables such as age and sex in this relationship. By addressing this gap in the literature, the study seeks to contribute to a more holistic and multidisciplinary approach to the assessment of patients with asthma. This study was conducted in Turkey, where asthma and fibromyalgia are both increasingly recognized as prevalent chronic conditions with substantial impact on daily functioning and quality of life. In the local clinical context, challenges such as underdiagnosis of fibromyalgia, limited interdisciplinary collaboration, and high variability in access to specialist care may influence how comorbid conditions are managed. These contextual factors informed the rationale for selecting a real-world, outpatient-based sample and underscore the importance of adopting multidimensional assessment approaches that go beyond respiratory symptoms alone. The study design thus reflects both the clinical realities and structural constraints of the national healthcare setting. Emerging evidence suggests shared mechanisms between asthma and fibromyalgia, including neurogenic inflammation mediated by cytokines (e.g. IL-6, TNF-α) and central sensitization. These overlapping pathways may explain the observed clinical comorbidity.

The hypotheses tested in this study are as follows:

1. The prevalence of fibromyalgia is higher in individuals with asthma compared to healthy controls.
2. The presence of fibromyalgia negatively affects asthma control and lowers ACT scores.
3. Individuals with fibromyalgia have a higher frequency of uncontrolled asthma.
4. The potential influence of demographic factors (age/sex) on this relationship will be explored.

## Methods

### Study design and participants

**Figure 1.**
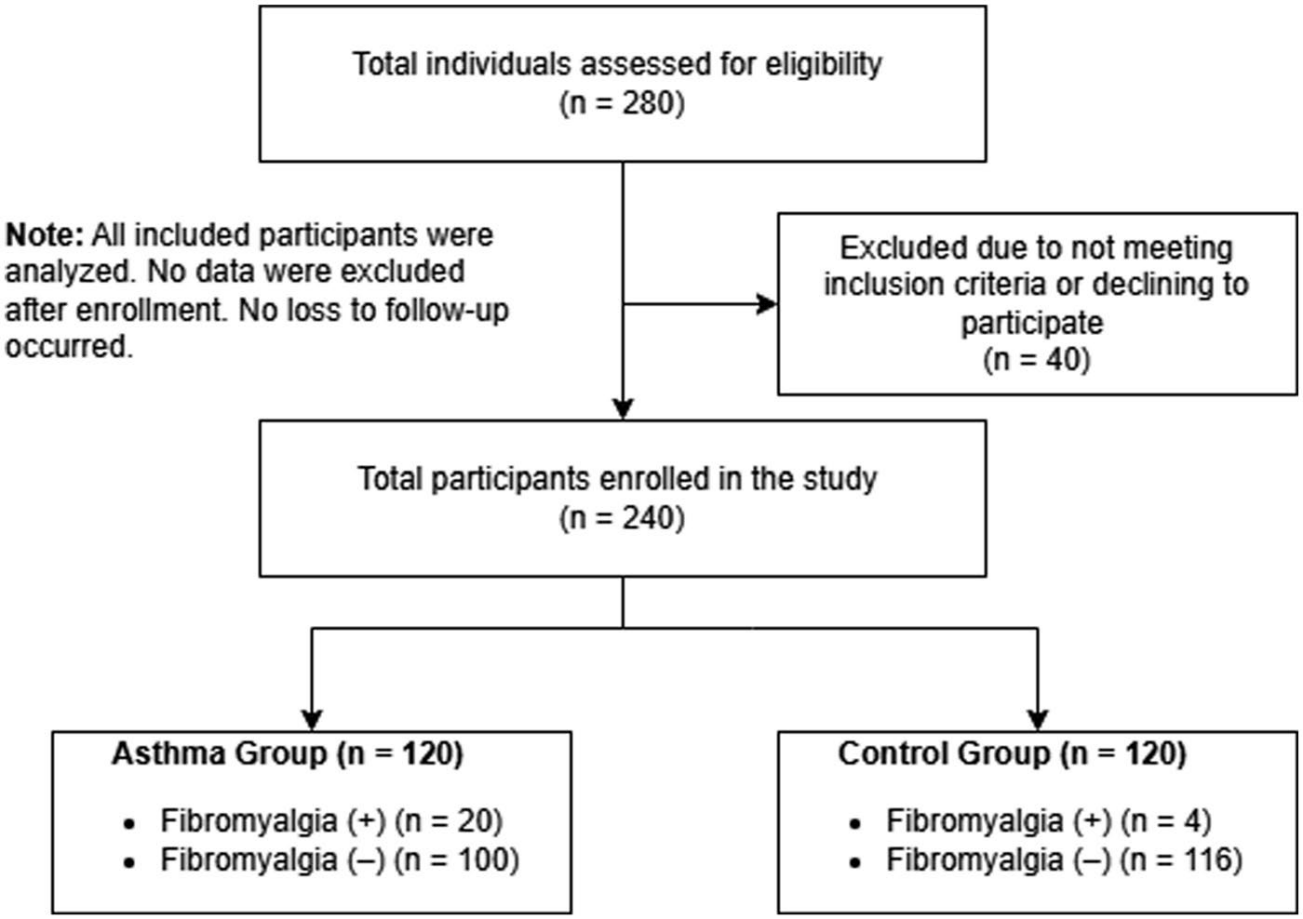
Flowchart of participant inclusion and group allocation.

This study was designed as a cross-sectional, case-control observational study conducted at the Chest Diseases Outpatient Clinic of a Sakarya university hospital in Turkey. Data were collected between January 2017 and June 2019. A total of 120 patients aged 18 to 65 years with a diagnosis of asthma based on the Global Initiative for Asthma (GINA) criteria were included, along with 120 age- and sex-matched healthy controls. The control group consisted of individuals without any diagnosed chronic illness and no self-reported respiratory complaints within the past year. Asthma patients were consecutively recruited from those attending the outpatient clinic during the study period, based on predefined eligibility criteria. To minimize selection bias and enhance comparability, age and sex matching was applied during group allocation, ensuring a representative sample reflective of real-world clinical practice. A convenience sampling strategy with consecutive recruitment was employed due to the clinical setting. While this approach enhances the feasibility and applicability to real-world practice, it does not ensure randomization. Therefore, the possibility of selection bias should be acknowledged when interpreting the results. To minimize bias, age and sex matching was applied during group selection. Validated tools were used for all assessments. Recall bias was reduced by limiting symptom evaluations to recent time periods (e.g. ACT: 4 weeks, fibromyalgia: 3 months). A priori power analysis was performed using G*Power 3.1.9.7 to determine the required sample size for detecting a difference in fibromyalgia prevalence between the asthma and control groups. Based on previous literature and pilot data, fibromyalgia prevalence was assumed to be 16.7% in the asthma group and 3.3% in the control group. With a two-tailed test, α = 0.05, power (1-β) = 0.80, and equal group sizes (allocation ratio = 1), the required sample size was calculated to be 69 participants per group (total *n* = 138). To account for potential dropouts and enhance statistical power, 120 individuals were recruited for each group. The participant enrollment process, including inclusion, exclusion, and final group allocation, is illustrated in Figure 1.

### Inclusion and exclusion criteria

#### Inclusion criteria

- Age between 18 and 65 years
- For the asthma group: a clinical diagnosis of asthma according to the GINA guidelines
- For the control group: no known chronic medical conditions and no history of respiratory complaints in the past 12 months
- Voluntary participation with signed informed consent
- Controls were additionally screened for other chronic pain disorders to minimize confounding.

#### Exclusion criteria

- Diagnosis of any rheumatologic disease, malignancy, neurological disorder, or active systemic infection
- History of severe cardiovascular disease or unstable medical conditions
- Presence of severe psychiatric illness (e.g., major depressive disorder, schizophrenia) or cognitive impairment affecting comprehension
- Current asthma exacerbation or acute respiratory infection
- Use of medications known to significantly affect pain perception or cognitive status within the past 3 months

### Assessment of fibromyalgia

Fibromyalgia was diagnosed according to the 2016 revised criteria of the ACR [14]. The diagnostic criteria included the Widespread Pain Index (WPI) and Symptom Severity Scale (SSS). Participants were classified as having fibromyalgia if they had WPI ≥ 7 and SSS ≥ 5, or WPI between 4 and 6 and SSS ≥ 9. These criteria were applied based on the symptoms reported over the previous three months.

### Assessment of asthma control

Asthma control was evaluated using the (ACT) [15], a self-administered questionnaire consisting of five items assessing symptoms and functional status over the past four weeks. The total score ranges from 5 to 25, with higher scores indicating better control. Asthma control levels were categorized as follows:

- Controlled asthma: ACT score ≥ 20
- Partially controlled asthma: ACT score between 16–19
- Uncontrolled asthma: ACT score ≤ 15

While ACT is validated for asthma control assessment, its focus on respiratory symptoms may limit accuracy in patients with systemic comorbidities like fibromyalgia.

### Ethical considerations

The study was approved by the Non-Interventional Research Ethics Committee of the Faculty of Medicine, Sakarya University (Protocol No: 71522473/050.01.04/59). Written informed consent was obtained from all participants. The study was conducted in accordance with the principles of the Declaration of Helsinki. All participants were consecutively recruited during routine visits to the outpatient clinic. Eligible individuals were verbally informed about the study objectives and procedures. Written informed consent was obtained prior to participation in accordance with the Declaration of Helsinki. To ensure participant confidentiality, all data were anonymized prior to analysis, and identifiable personal information was removed from the datasets. No individual-level data are reported in this manuscript. Data safeguarding protocols were implemented in accordance with the approval of the Research Ethics Committee and the Law on the Protection of Personal Data.

### Statistical analysis

Statistical analyses were conducted using IBM SPSS Statistics for Windows, Version 25.0 (IBM Corp., Armonk, NY, USA). Continuous variables were expressed as mean ± standard deviation (SD), while categorical variables were presented as frequencies and percentages. The independent samples t-test was used to compare normally distributed continuous variables between groups. The chi-square test (χ^2^) was used for the analysis of categorical variables.

To assess the association between fibromyalgia and uncontrolled asthma, logistic regression analysis was performed, adjusting for age and sex as covariates. Results were reported as odds ratios (OR) with 95% confidence intervals (CI). To assess the relationship between fibromyalgia and asthma control, a binary logistic regression analysis was conducted. The dependent variable was asthma control status, dichotomized as uncontrolled or partially controlled asthma (coded as 1) versus controlled asthma (coded as 0). The primary independent variable was the presence of fibromyalgia (yes = 1, no = 0). The model was adjusted for age and sex. Odds ratios (ORs) with 95% confidence intervals (CIs) were calculated. An OR > 1 was interpreted as an increased likelihood of uncontrolled asthma.

In addition, the correlation between fibromyalgia symptom severity (WPI and SSS scores) and ACT score was examined using Pearson’s correlation coefficient (r). The ability of the ACT score to discriminate between participants with and without fibromyalgia was evaluated using receiver operating characteristic (ROC) curve analysis, and the area under the curve (AUC) was calculated. A p-value of < 0.05 was considered statistically significant in all analyses.

Descriptive statistics were presented as means ± standard deviation for continuous variables and as frequencies and percentages for categorical variables. Group comparisons were conducted using the chi-square test for categorical variables and the independent-samples t-test or Mann-Whitney U test for continuous variables, depending on data distribution.

To evaluate the association between fibromyalgia and asthma control, a binary logistic regression analysis was performed. The presence of fibromyalgia (yes/no) was entered as the dependent variable. Independent variables included demographic and clinical parameters such as age, sex, body mass index (BMI), duration of asthma, comorbidities, and ACT score. Crude and adjusted odds ratios (OR) with 95% confidence intervals (CI) were calculated.

In addition, the correlation between fibromyalgia symptom severity measured by Widespread Pain Index (WPI) and Symptom Severity Scale (SSS) scores and ACT score was examined using Pearson’s correlation coefficient (r). The ability of the ACT score to discriminate between participants with and without fibromyalgia was evaluated using receiver operating characteristic (ROC) curve analysis, and the area under the curve (AUC) was calculated. All analyses were conducted using SPSS version 25.0 (IBM Corp., Armonk, NY, USA), and the significance level was set at *p* < 0.05.

## Results

### Participant characteristics and fibromyalgia prevalence

A total of 240 participants were included in the study, with 120 individuals in the asthma group and 120 in the control group. The mean age was 45.3 ± 9.9 years in the asthma group and 44.7 ± 9.5 years in the control group, with no statistically significant difference between the groups (*p* = 0.76). The gender distribution was similar in both groups, with 60% of participants being female and 40% male (*p* = 0.82). Subgroup analysis by sex showed fibromyalgia prevalence of 18.3% in female vs. 12.5% in male asthma patients (*p* = 0.21). Age-stratified analysis revealed no significant trends.

The prevalence of fibromyalgia was 16.7% (*n* = 20) in the asthma group and 3.3% (*n* = 4) in the control group. This difference was statistically significant (χ^2^ = 11.25, *p* < 0.001). The odds ratio for the coexistence of asthma and fibromyalgia was calculated as 5.83 (95% CI: 1.94–17.54). The ACT showed no discriminative capacity for fibromyalgia (AUC = 0.50, equivalent to random chance).

### ACT score and asthma control

The mean ACT score was 18.2 ± 2.9 in asthma patients with fibromyalgia and 20.1 ± 2.4 in those without fibromyalgia. This difference was statistically significant (*t* = 2.94, *p* = 0.004).

The distribution of asthma control levels according to the presence of fibromyalgia is presented in Table 1. Among patients with fibromyalgia, 60% had uncontrolled asthma and 40% had partially controlled asthma. In contrast, among those without fibromyalgia, 35% had uncontrolled asthma and 24% had partially controlled asthma. This distribution showed a statistically significant difference (*p* = 0.03, chi-square test).

**Table 1.** Distribution of asthma control levels according to the presence of fibromyalgia in the asthma group.

Presence of Fibromyalgia?	Controlled	Partially Controlled	Uncontrolled
Yes (*n* = 20)	3	8	9
No (*n* = 100)	41	24	35

**Figure 2.**
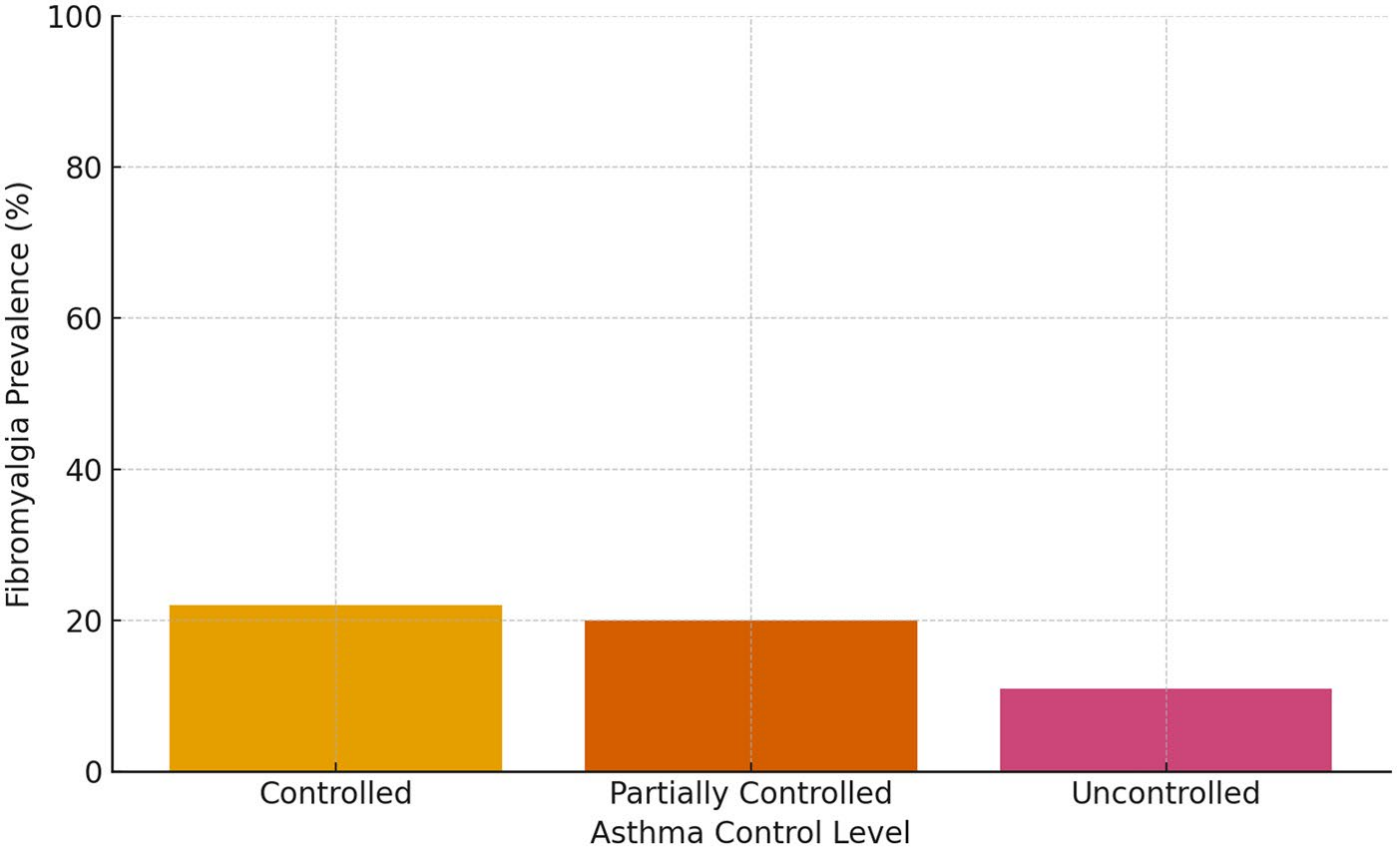
Prevalence of fibromyalgia across different levels of asthma control.

The distribution of asthma control levels according to the presence of fibromyalgia is presented in Table 1. Among individuals diagnosed with fibromyalgia, 60% were classified as having uncontrolled asthma and 40% as partially controlled. In comparison, among those without fibromyalgia, 35% had uncontrolled asthma and 24% had partially controlled asthma. This distribution was statistically significant (*p* = 0.03, chi-square test). Figure 2 visually illustrates how the prevalence of fibromyalgia varies across different levels of asthma control.

### Logistic regression analysis

To assess the association between fibromyalgia and asthma control, a binary logistic regression analysis was conducted. The dependent variable was asthma control status, dichotomized as uncontrolled or partially controlled asthma (coded as 1) versus controlled asthma (coded as 0). The presence of fibromyalgia was included as the main independent variable, and the model was adjusted for age and sex. The coding was performed such that an odds ratio (OR) greater than 1 indicates an increased likelihood of uncontrolled asthma. In this model, fibromyalgia was associated with higher odds of having uncontrolled asthma (OR = 2.17, 95% CI: 0.85–5.51; *p* = 0.103), although this association did not reach statistical significance. Neither age nor sex was a statistically significant predictor (*p* > 0.05 for both) (Table 2).

**Table 2.** Logistic regression analysis for predicting uncontrolled asthma.

Variable	Coefficient (B)	OR (95% CI)	p-value
Fibromyalgia	0.770	2.16 (0.74–6.55)	0.103
Age	0.003	1.00 (0.96–1.04)	0.899
Gender (Male)	0.250	1.28 (0.60–2.75)	0.524

A binary logistic regression analysis was conducted to examine whether the presence of fibromyalgia predicts poor asthma control. The presence of fibromyalgia was associated with higher odds of having uncontrolled or partially controlled asthma compared to controlled asthma (OR = 2.17, 95% CI: 0.85–5.51; *p* = 0.103), although this association did not reach statistical significance. Neither age nor sex was a statistically significant predictor (*p* > 0.05 for both) (Table 2).

### Relationship between fibromyalgia symptom severity and ACT score

Pearson correlation analyses were conducted to examine the relationship between ACT scores and WPI and SSS scores in asthma patients. Both correlations were found to be weak and statistically non-significant:

- ACT vs. WPI: *r* = –0.014, *p* = 0.882
- ACT vs. SSS: *r* = +0.091, *p* = 0.323

**Figure 3.**
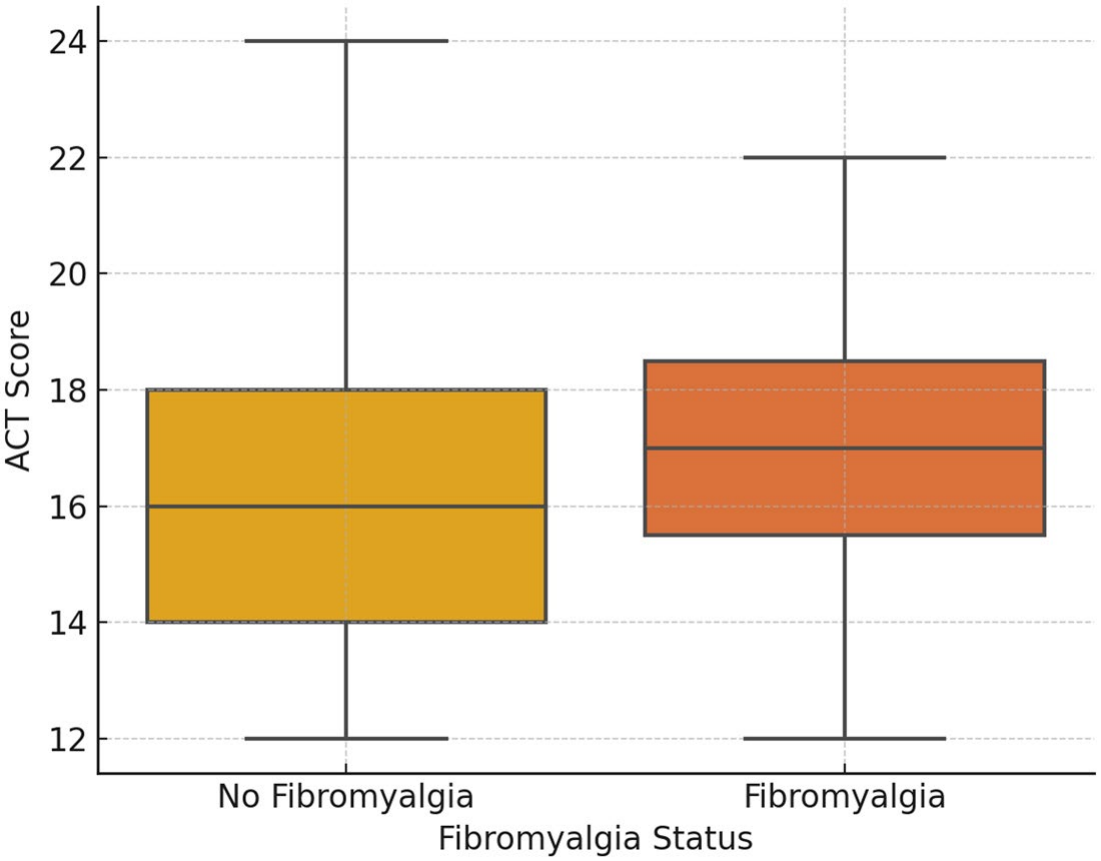
Comparison of ACT scores between asthma patients with and without fibromyalgia.

The median ACT score was lower in patients with fibromyalgia compared to those without, supporting the potential negative impact of fibromyalgia on asthma control (Figure 3).

These findings indicate that fibromyalgia symptom severity does not directly influence ACT scores, although a diagnostic-level distinction is present.

### Discriminative ability of ACT score for fibromyalgia diagnosis

**Figure 4.**
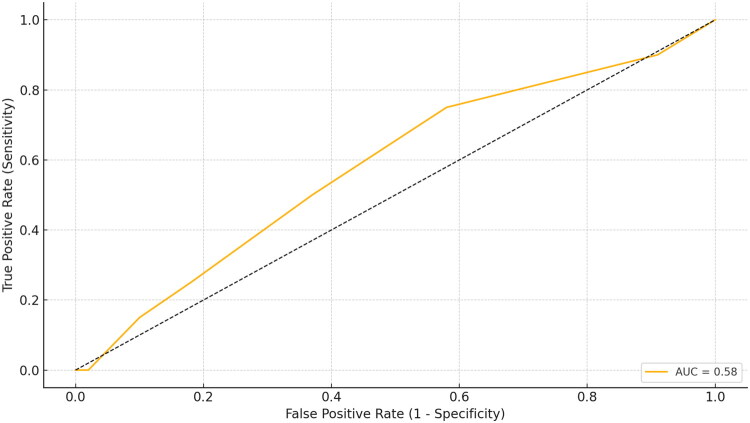
Receiver operating characteristic (ROC) curve for the discriminative ability of ACT score to identify fibromyalgia.

The performance of the ACT score in predicting fibromyalgia diagnosis was assessed using a ROC curve. The analysis yielded an AUC value of 0.50. This result indicates that the ACT score has no discriminative power beyond chance in identifying fibromyalgia (Figure 4). Therefore, the ACT is not a suitable screening tool for detecting fibromyalgia.

## Discussion

This study provides evidence supporting the high comorbidity of fibromyalgia in asthma and its potential impact on asthma control. Our findings show that ACT scores were significantly lower in patients with fibromyalgia, although this was not reflected in logistic regression as a statistically significant predictor. Our findings support those of Gorial et al. (2020), who reported that approximately 30% of asthma patients were diagnosed with fibromyalgia [4]. Previous studies have reported fibromyalgia prevalence in asthma patients ranging from 3% to 31%, and the 16.7% prevalence identified in our study falls within this range [16,17].

In our study, age and sex were not found to be independent predictors of the relationship between fibromyalgia and asthma control. This suggests that the influence of demographic variables such as age and sex may be limited in asthma patients with comorbid fibromyalgia. The literature on this topic presents conflicting findings; while some studies suggest that female sex is a risk factor for fibromyalgia [16], others have reported no significant sex-related differences in fibromyalgia prevalence [18].

While the presence of fibromyalgia was associated with significantly lower ACT scores, ROC analysis revealed that the ACT had poor discriminative power for detecting this comorbidity (AUC = 0.50). Contrary to initial hypotheses, demographic factors did not significantly modify the fibromyalgia-asthma relationship. This may reflect the homogeneous distribution of fibromyalgia symptoms across age/sex groups in our cohort, or limited power to detect subtle effects. Although logistic regression did not identify fibromyalgia as a statistically significant independent predictor of uncontrolled asthma, the direction of the association (OR > 1) was consistent with our hypothesis that fibromyalgia may worsen asthma control. This suggests that the ACT, which focuses solely on respiratory symptoms, may fail to account for systemic comorbidities such as fibromyalgia.

Although fibromyalgia and asthma affect different physiological systems, they share common pathophysiological mechanisms through inflammatory processes, central sensitization, and psychosocial factors [11,19]. Elevated levels of proinflammatory mediators such as substance P, interleukin-6 (IL-6), and C-reactive protein (CRP) have been observed in both conditions, biologically supporting the coexistence of asthma and fibromyalgia [12,20–22]. Central sensitization is considered a key mechanism in the overlap between the two disorders. Unexplained symptom severity and heightened pain perception seen in fibromyalgia have similarly been linked to central sensitization processes in asthma [11,23]. Previous literature suggests that neurogenic inflammation enhances pain perception in the central nervous system among fibromyalgia patients and contributes to airway hyperresponsiveness in individuals with asthma [7,24].

This shared inflammatory substrate may amplify symptom burden and negatively impact quality of life [4,13,25]. Symptoms that are commonly observed in fibromyalgia, such as fatigue, sleep disturbances, and widespread pain, can intensify asthma-related symptoms and may cause patients to report lower scores on self-reported assessment tools like the ACT [13]. These findings align with our observation that ACT scores were significantly lower among asthma patients diagnosed with fibromyalgia. The notably reduced ACT scores in these individuals suggest that systemic symptoms related to fibromyalgia may distort self-reported asthma control outcomes [26]. However, the ACT’s inability to capture these non-respiratory symptoms reduces its diagnostic sensitivity and highlights the need for more comprehensive assessment instruments [13].

Psychological comorbidities further complicate the interaction between fibromyalgia and asthma. High rates of depression and anxiety are commonly observed in patients with both conditions, negatively affecting disease control and treatment adherence [9,10]. The characteristic cognitive symptoms of fibromyalgia, often referred to as “fibro fog,” may also interfere with practical aspects of asthma management, such as adherence to inhaler use [27]. Clinically, the presence of fibromyalgia complicates asthma management by increasing healthcare utilization, promoting polypharmacy, and significantly reducing quality of life [25,28,29]. Therefore, rather than relying solely on traditional approaches focused on respiratory symptoms, a multidisciplinary model that includes physical therapy, psychosocial support, and cognitive behavioral therapy is recommended [30,31]. Additionally, although certain nonsteroidal anti-inflammatory drugs (NSAIDs) are commonly used in the management of fibromyalgia, caution is required in asthma patients due to the potential risk of NSAID-exacerbated respiratory disease (AERD) [32].

Our study suggests that fibromyalgia is not only a common comorbidity in asthma patients but may also have an independent impact on asthma control. Weatherburn et al. [33] and Calvo-Lobo et al. [34] have reported that migraine and widespread pain are frequently observed in individuals with asthma and may be linked to central sensitization mechanisms. These findings support the neurophysiological basis of the symptom overlap demonstrated in our study. A multicenter case-control study by Martínez-Moragón et al. [25] identified fibromyalgia as a significant contributing factor to uncontrolled asthma. This finding reinforces our hypothesis that fibromyalgia should be regarded not merely as a coexisting condition, but also as an independent risk factor affecting asthma control.

Accordingly, in individuals with both conditions, treatment plans should not only include bronchodilators and anti-inflammatory therapies but also incorporate strategies for pain management and psychological support [10]. From a therapeutic perspective, a multidisciplinary approach is essential when asthma and fibromyalgia coexist. While the use of biologic agents in asthma treatment is increasing, the management of fibromyalgia typically involves physical activity, antidepressants, and cognitive behavioral therapy [35]. However, because fibromyalgia may limit patients’ ability to engage in physical exercise, exercise prescriptions should be individualized and implemented under the supervision of a physiotherapist when necessary [36].

In our study, a ROC analysis was conducted to evaluate the discriminative ability of the ACT score in identifying the presence of fibromyalgia. The area AUC was found to be 0.50, indicating that the ACT performs at chance level in detecting fibromyalgia and is therefore inadequate as a diagnostic tool. On the other hand, the significantly lower ACT scores observed in individuals diagnosed with fibromyalgia suggest that this comorbidity may negatively influence asthma control. Taken together, these findings imply that low ACT scores may be indicative of an increased risk of fibromyalgia; however, it does not appear feasible to define a specific diagnostic threshold based on ACT scores.

The results emphasize the need for more holistic and multidimensional approaches rather than relying solely on ACT scores when assessing asthma control in clinical practice. Particularly in patients with suboptimal asthma control, routine screening for fibromyalgia may help avoid unnecessary treatment intensification and support a more personalized approach to disease management. Early diagnosis of fibromyalgia not only helps avoid unnecessary escalation of asthma treatment but also facilitates timely management of associated symptoms such as chronic pain, sleep disturbances, and depression, ultimately improving overall quality of life. As highlighted in previous research, delayed diagnosis may lead to reduced treatment adherence and increased healthcare utilization [37]. Thus, incorporating fibromyalgia screening into routine asthma care is essential for delivering more comprehensive and individualized management strategies.

### Strengths and limitations

Among the strengths of our study are the use of up-to-date and validated diagnostic criteria for fibromyalgia, namely the 2016 ACR criteria, and the assessment of asthma control using the ACT, a widely accepted and validated instrument. In addition, matching cases and controls for potential confounding variables such as age and sex enhances the internal validity of the findings.

However, the study has several limitations. First, its single-center design and relatively small sample size may limit the generalizability of the results. The cross-sectional design precludes causal inference about the fibromyalgia-asthma relationship. As participants were recruited using a non-random consecutive sampling strategy, the findings may be subject to selection bias, potentially limiting the generalizability of the results. Furthermore, important psychosocial variables such as depression, anxiety, and quality of life were not assessed; these factors could potentially influence both asthma control and fibromyalgia diagnosis.

Additionally, although fibromyalgia was diagnosed using clinical criteria based on WPI and SSS scores, no objective biomarkers were used to support the diagnosis. This may limit diagnostic certainty. Since the ACT is a self-reported tool, symptoms such as fatigue, pain, and depression associated with fibromyalgia may bias patient responses and artificially lower ACT scores, reducing the test’s specificity. ACT is designed to focus on respiratory symptoms alone and does not account for comorbid conditions with systemic symptoms like fibromyalgia. This limitation should be considered when interpreting ACT scores, as it may affect the accuracy of asthma control assessment in such patients.

Another limitation of our study is the reliance on self-reported measures for both asthma control (ACT) and fibromyalgia symptoms (WPI and SSS), which may introduce self-reporting bias. Symptoms such as pain, fatigue, or shortness of breath may be subjectively influenced by mood, perception, or cognitive status. Moreover, while we used validated clinical criteria to diagnose fibromyalgia, no standardized psychological assessments (e.g. for depression or anxiety) were conducted. The absence of such evaluations may limit the interpretability of symptom overlap between the two conditions.

The limited discriminative power of the ACT observed in our study underscores the need for incorporating fibromyalgia screening and multidimensional assessment methods into clinical practice. Particularly in patients with suboptimal asthma control, routine screening for fibromyalgia may prevent unnecessary treatment intensification and contribute to more personalized disease management.

Finally, the cross-sectional design of the study precludes causal inference. Nonetheless, our findings suggest that the presence of fibromyalgia may negatively impact asthma control and highlight the importance of integrating fibromyalgia screening into routine clinical evaluations. Specifically, screening for fibromyalgia in asthma patients presenting with chronic pain and fatigue may lead to more comprehensive and effective care strategies.

### Implications for practice and research

The findings of this study indicate that the ACT, when used in isolation, may not adequately detect comorbid conditions such as fibromyalgia. In clinical practice, it is essential to consider not only respiratory symptoms but also systemic and psychological factors when assessing asthma control. Therefore, the integration of multidimensional assessment tools or comorbidity screening protocols into routine asthma follow-up is strongly recommended.

From a research perspective, there is a need for advanced studies to explore the interactions between ACT scores and various comorbid conditions in greater depth. Randomized controlled trials, in particular, would be valuable in evaluating the impact of fibromyalgia management on asthma control. Furthermore, investigating underlying pathophysiological mechanisms through biomarkers may contribute to the development of more comprehensive and personalized treatment strategies.

## Conclusion

This study highlights that the presence of fibromyalgia should not be overlooked in patients with asthma. The significantly higher prevalence of fibromyalgia among individuals with asthma, along with their lower levels of asthma control, suggests a clinically meaningful interaction between the two conditions. Fibromyalgia is a common comorbidity in asthma patients and may adversely affect asthma control. However, our findings indicate that demographic factors such as age and sex are not independent determinants of this relationship. These results underscore the importance of incorporating fibromyalgia screening into asthma management and support the adoption of multidisciplinary approaches to enhance patient outcomes. Further studies with larger sample sizes and multicenter, prospective designs are warranted to clarify the direction and underlying mechanisms of this association.

## Supplementary Material

STROBE checklist case control.doc

Figure Captions.docx

## Data Availability

Data and materials are available upon request to the corresponding author.

## References

[1] Global Initiative for Asthma (GINA). Global strategyfor asthma management and prevention; 2023. Available from: https://ginaasthma.org/.

[2] Wolfe F, Clauw DJ, Fitzcharles MA, et al. The American College of Rheumatology preliminary diagnostic criteria for fibromyalgia and measurement of symptom severity. Arthritis Care Res (Hoboken). 2010;62(5):600–610. doi: 10.1002/acr.20140. 20461783

[3] Galvez-Sánchez CM, Reyes del Paso GA, Montoro CI. Revealing the role of social support on cognitive deficits in fibromyalgia syndrome. Behav Neurol. 2022;2022(1):3852746. doi: 10.1155/2022/3852746. 36091221 PMC9458397

[4] Gorial FI, Allawerdi MA, Abd Ali MN. Fibromyalgia in Iraqi patients with asthma and its impact on asthma severity and control. Ann Med Surg (Lond). 2020;60:22–26. doi: 10.1016/j.amsu.2020.10.019. 33101668 PMC7575834

[5] Rodríguez‐Torres J, López‐López L, Cabrera‐Martos I, et al. Symptom severity is associated with signs of central sensitization in patients with asthma. Clin Respir J. 2021;15(11):1219–1226. doi: 10.1111/crj.13429. 34328269

[6] Fleming KC, Volcheck MM. Central sensitization syndrome and the initial evaluation of a patient with fibromyalgia: a review. Rambam Maimonides Med J. 2015;6(2):e0020. doi: 10.5041/RMMJ.10204. 25973272 PMC4422459

[7] Bekdas M, Saygi B, Kilinc YB, et al. Plasma levels of neurogenic inflammation related neuropeptides in pediatric patients with community-acquired pneumonia and their potential diagnostic value in distinguishing viral and bacterial pneumonia. Eur J Pediatr. 2024;183(4):1619–1627. doi: 10.1007/s00431-023-05417-y. 38183438 PMC11001734

[8] Arnold LM. The pathophysiology, diagnosis and treatment of fibromyalgia. Psychiatr Clin North Am. 2010;33(2):375–408. doi: 10.1016/j.psc.2010.01.001. 20385343

[9] Akula M, Kulikova A, Khan DA, et al. The relationship between asthma and depression in a community-based sample. J Asthma. 2018;55(12):1271–1277. doi: 10.1080/02770903.2017.1418885. 29336633 PMC6212321

[10] Arnold LM, Hudson JI, Keck PE, et al. Comorbidity of fibromyalgia and psychiatric disorders. J Clin Psychiatry. 2006;67(8):1219–1225. doi: 10.4088/jcp.v67n0807. 16965199

[11] Hyland ME, Lanario JW, Wei Y, et al. Evidence for similarity in symptoms and mechanism: the extra-pulmonary symptoms of severe asthma and the polysymptomatic presentation of fibromyalgia. Immun Inflamm Dis. 2019;7(4):239–249. doi: 10.1002/iid3.263. 31441602 PMC6842811

[12] Tsilioni I, Russell IJ, Stewart JM, et al. Neuropeptides CRH, SP, HK-1, and inflammatory cytokines IL-6 and TNF are increased in serum of patients with fibromyalgia syndrome, implicating mast cells. J Pharmacol Exp Ther. 2016;356(3):664–672. doi: 10.1124/jpet.115.230060. 26763911 PMC4767394

[13] de Felice G, Hyland ME, Lanario JW, et al. Preliminary development of a questionnaire to measure the extra-pulmonary symptoms of severe asthma. BMC Pulm Med. 2021;21(1):369. doi: 10.1186/s12890-021-01730-0. 34775957 PMC8591792

[14] Wolfe F, Clauw DJ, Fitzcharles MA, et al. Wallitt Brian. 2016 Revisions to the 2010/2011 fibromyalgia diagnostic criteria. Semin Arthritis Rheum. 2016;46(3):319–329. doi: 10.1016/j.semarthrit.2016.08.012. 27916278

[15] Nathan RA, Sorkness CA, Kosinski M, et al. Development of the asthma control test: a survey for assessing asthma control. J Allergy Clin Immunol. 2004;113(1):59–65. doi: 10.1016/j.jaci.2003.09.008. 14713908

[16] Heidari F, Afshari M, Moosazadeh M. Prevalence of fibromyalgia in general population and patients, a systematic review and meta-analysis. Rheumatol Int. 2017;37(9):1527–1539. doi: 10.1007/s00296-017-3725-2. 28447207

[17] Darçın T, Köseoğlu HK. Evaluation of metabolic syndrome prevalence and parameters in patients with fibromyalgia. Anatol J Family Med. 2020;3(2):167–172. doi: 10.5505/anatoljfm.2020.41636.

[18] Queiroz LP. Worldwide epidemiology of fibromyalgia. Curr Pain Headache Rep. 2013;17(8):356. doi: 10.1007/s11916-013-0356-5. 23801009

[19] Xu Z, Wang F, Adekkanattu P, et al. Subphenotyping depression using machine learning and electronic health records. Learn Health Syst. 2020;4(4):e10241. doi: 10.1002/lrh2.10241. 33083540 PMC7556423

[20] Fairweather D, Bruno KA, Darakjian AA, et al. High overlap in patients diagnosed with hypermobile Ehlers-Danlos syndrome or hypermobile spectrum disorders with fibromyalgia and 40 self-reported symptoms and comorbidities. Front Med (Lausanne). 2023;10:1096180. doi: 10.3389/fmed.2023.1096180. 37181352 PMC10166812

[21] Fujita Y, Matsuoka H, Chiba Y, et al. Novel single nucleotide polymorphism biomarkers to predict opioid effects for cancer pain. Oncol Lett. 2023;26(2):355. doi: 10.3892/ol.2023.13941. 37545623 PMC10398630

[22] Poynter ME, Irvin CG. Interleukin-6 as a biomarker for asthma: hype or is there something else? Eur Respir J. 2016;48(4):979–981. doi: 10.1183/13993003. 27694408 PMC5518110

[23] Mezhov V, Guymer E, Littlejohn G. Central sensitivity and fibromyalgia. Intern Med J. 2021;51(12):1990–1998. doi: 10.1111/imj.15430. 34139045

[24] Miecznikowski W, Mielcarska S, Kiczmer P, et al. Changes of substance P, NGF and CGRP salivary levels among patients undergoing physical therapy. Med Res J. 2020;5(4):238–243. doi: 10.5603/MRJ.a2020.0041.

[25] Martinez-Moragon E, Plaza V, Torres I, et al. Fibromyalgia as a cause of uncontrolled asthma: a case–control multicenter study. Curr Med Res Opin. 2017;33(12):2181–2186. doi: 10.1080/03007995.2017.1354828. 28699806

[26] Janevic MR, Ellis KR, Sanders GM, et al. Self-management of multiple chronic conditions among African American women with asthma: a qualitative study. J Asthma. 2014;51(3):243–252. doi: 10.3109/02770903.2013.860166. 24161047 PMC4234073

[27] Choy E, Perrot S, Leon T, et al. A patient survey of the impact of fibromyalgia and the journey to diagnosis. BMC Health Serv Res. 2010;10(1):102. doi: 10.1186/1472-6963-10-102. 20420681 PMC2874550

[28] Calderón-Larrañaga A, Gimeno-Feliu LA, González-Rubio F, et al. Polypharmacy patterns: unravelling systematic associations between prescribed medications. PLoS One. 2013;8(12):e84967. doi: 10.1371/journal.pone.0084967. 24376858 PMC3869920

[29] Nishikawara RK, Schultz IZ, Butterfield LD, et al. You have to believe the patient: what do people with fibromyalgia find helpful (and hindering) when accessing health care? Can J Pain. 2023;7(2):2176745. doi: 10.1080/24740527.2023.2176745. 37025116 PMC10072062

[30] Wu J, Chen Z, Zheng K, et al. Benefits of exergame training for female patients with fibromyalgia: a systematic review and meta-analysis of randomized controlled trials. Arch Phys Med Rehabil. 2022;103(6):1192–1200. e2. doi: 10.1016/j.apmr.2021.10.022. 35033538

[31] Castro-Piñero J, Aparicio VA, Estévez-López F, et al. The potential of established fitness cut-off points for monitoring women with fibromyalgia: the Al-Ándalus Project. Int J Sports Med. 2017;38(5):359–369. doi: 10.1055/s-0043-101912. 28315284

[32] Tan JHY, Hsu AAL. Nonsteroidal anti-inflammatory drug (NSAID) exacerbated respiratory disease phenotype: topical NSAID and asthma control – A possible oversight link. Respir Med. 2016;118:1–3. doi: 10.1016/j.rmed.2016.07.004. 27578463

[33] Weatherburn CJ, Guthrie B, Mercer SW, et al. Comorbidities in adults with asthma: population‐based cross‐sectional analysis of 1.4 million adults in Scotland. Clin Exp Allergy. 2017;47(10):1246–1252. doi: 10.1111/cea.12971. 28665552

[34] Calvo-Lobo C, Painceira-Villar R, López-López D, et al. Tarsal tunnel mechanosensitivity is increased in patients with asthma: a case-control study. J Clin Med. 2018;7(12):541. doi: 10.3390/jcm7120541. 30545067 PMC6306873

[35] Rogliani P, Calzetta L, Matera MG, et al. Severe asthma and biological therapy: when, which, and for whom. Pulm Ther. 2020;6(1):47–66. doi: 10.1007/s41030-019-00109-1. 32048241 PMC7229123

[36] Antunes MD, Marques AP. The role of physiotherapy in fibromyalgia: current and future perspectives. Front Physiol. 2022;13:968292. doi: 10.3389/fphys.2022.968292. 36051912 PMC9424756

[37] Qureshi AG, Jha SK, Iskander J, et al. Diagnostic challenges and management of fibromyalgia. Cureus. 2021;13(10):e18692. doi: 10.7759/cureus.18692. 34786265 PMC8580749

