# Supplementary material for: Prevalence of fibromyalgia in asthma and its impact on asthma control: a case-control study
Source: Ann Med. 2025 Sep 29;57(1):2566392. doi: 10.1080/07853890.2025.2566392 (PMC12481540; doi:10.1080/07853890.2025.2566392)
Supplement: Figure Captions.docx [file IANN_A_2566392_SM4829.docx]

**Figure Captions**

**Figure 1.** Flow chart of the participant selection process.

**Figure 2.** Prevalence of fibromyalgia across asthma control levels.

**Figure 3.** ACT score distribution by fibromyalgia status.

**Figure 4.** ROC curve for the ACT score in predicting fibromyalgia.
